# A Case of Elbow Extension Limitation Caused by Phlebosclerosis Following Peripheral Intravenous Chemotherapy: A Case Report and Literature Review

**DOI:** 10.3400/avd.cr.25-00055

**Published:** 2025-07-01

**Authors:** Nozomu Ishikawa, Kazunori Inuzuka, Masaki Sano, Kazuto Katahashi, Hajime Tsuyuki, Yusuke Endo, Hiroya Takeuchi, Naoki Unno

**Affiliations:** 1 Division of Vascular Surgery, Hamamatsu University School of Medicine, Hamamatsu, Shizuoka, Japan; 2 Second Department of Surgery, Hamamatsu University School of Medicine, Hamamatsu, Shizuoka, Japan; 3 Department of Vascular Surgery, Hamamatsu Medical Center, Hamamatsu, Shizuoka, Japan

**Keywords:** phlebosclerosis, elbow contracture, chemotherapy

## Abstract

We report a rare case of phlebosclerosis in the left antecubital region after peripheral intravenous chemotherapy, which caused elbow contracture. A 54-year-old woman with breast cancer underwent partial mastectomy of the right breast and 8 courses of neoadjuvant chemotherapy, 6 of which were administered via venipuncture in the left forearm. She developed progressive flexion contracture of the left elbow despite rehabilitation. Two cord-like fibrotic veins were identified in the antecubital region and surgically excised. Postoperative rehabilitation led to full elbow extension within 5 months. In cases with elbow extension limitation, proactive surgical intervention should be considered.

## Introduction

When chemotherapy is administered via peripheral veins, pain, erythema, and warmth due to phlebitis (thrombophlebitis) often occur.^1)^ However, in rare cases, phlebosclerosis may develop, leading to the formation of cord-like structures, which can cause joint extension limitation.^2)^ Here, we report an extremely rare case of phlebosclerosis originating in the elbow, which led to elbow extension limitation, along with a review of the literature.

## Case Report

**Figure 1:**
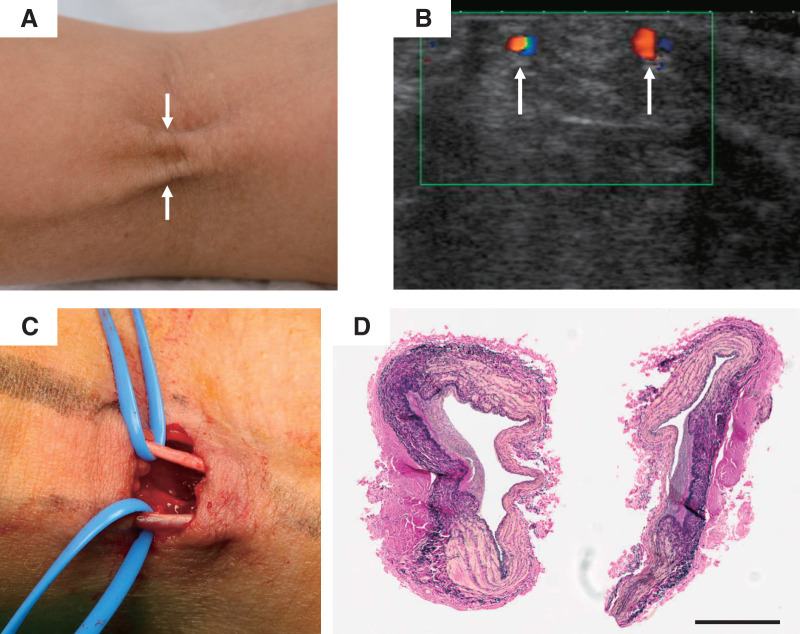
Fig. 1 Perioperative findings. (**A**) Two cord-like structures in the left elbow (arrows). Left is distal (wrist side), right is proximal (shoulder side). (**B**) Ultrasound findings of veins in the antecubital region. Blood flow within the lumen confirmed by forearm milking maneuver (Doppler ultrasound image, arrows). (**C**) Intraoperative findings: Two cord-like structures (cutaneous veins) in the antecubital region. Left is distal (wrist side), right is proximal (shoulder side). (**D**) Histopathological examination of the resected venous specimens of each cord-like structure was performed using Elastica van Gieson staining. Scale bar = 500 μm. Thickening of the intima and narrowing of the lumen, disruption of the smooth muscle layer in the media, and proliferation of elastic fibers and collagen. No thrombosis was observed in either vein.

A 54-year-old woman was diagnosed with right breast cancer (T2N1M0, Stage IIB). She underwent 4 courses of chemotherapy with cyclophosphamide and epirubicin, followed by 4 courses of trastuzumab, pertuzumab, and docetaxel. Of the 8 total chemotherapy courses, 6 were administered via venipuncture in the left forearm. Six months after the initiation of chemotherapy, the patient underwent partial mastectomy of the right breast along with tailored axillary surgery. Approximately 1 month later, she began experiencing limited range of motion in her left elbow, with an extension deficit of −10°, which corresponds to approximately 7 months after the initial chemotherapy. The final chemotherapy session was administered 5 months after the 1st, about 2 months before the onset of the contracture. According to the medical records, 3 weeks after starting the 1st course of chemotherapy, the patient reported that she had experienced pain in the vein at the venipuncture site. Rehabilitation therapy for the elbow joint was initiated, but the condition progressed to a flexion contracture with an extension deficit of −70°, and the patient was subsequently referred to orthopedic surgery. Two cord-like structures were observed in the left antecubital region, prompting a referral to our vascular surgery department (**Fig. 1A**).

Ultrasound examination revealed that the cord-like structures were thickened veins with preserved blood flow (**Fig. 1B**), but the distal forearm cutaneous veins were occluded due to thrombosis. Our vascular surgery team recommended excision of the cord-like structures, but the patient preferred to continue rehabilitation therapy for a while longer, which resulted in a delay before surgical intervention. Seven months after breast cancer surgery, the 2 thickened cord-like veins were ligated and resected under local anesthesia (**Fig. 1C**).

**Figure 2:**
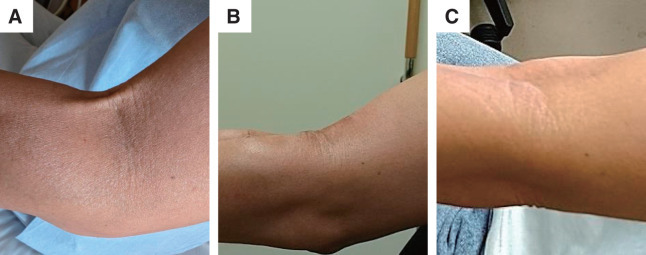
Fig. 2 Course of elbow extension. (**A**) Preoperative limitation of left elbow extension to −70° due to cord-like structures. (**B**) One month postoperatively, with elbow extension limited to −20°. (**C**) Five months postoperatively, with recovery of elbow extension to 5°.

The pathological findings of both cord-like veins revealed thickening of the intima and narrowing of the lumen, disruption of the smooth muscle layer in the media, and proliferation of elastic fibers and collagen. No thrombosis was observed in either vein. These findings are consistent with those of phlebosclerosis (**Fig. 1D**). Postoperatively, the elbow extension improved to a deficit of −45°. However, significant tension and shortening of the biceps brachii persisted. The patient continued with rehabilitation therapy for joint range-of-motion training, and 5 months after the venous resection, she achieved complete extension of the elbow, with a final extension of 5° (**Fig. 2**).

## Discussion

Chemical irritation caused by chemotherapy commonly leads to phlebitis/thrombotic phlebitis, often presenting with symptoms of acute inflammation such as redness, pain, and swelling.^3,4)^ Additionally, a palpable venous cord may form, but it is rare for this to cause functional impairment.^5)^ The general treatment methods include the application of warm compresses and the administration of nonsteroidal anti-inflammatory drugs.^6,7)^ However, the development of phlebosclerosis in the cubital vein, leading to restriction of elbow extension as seen in this case, is extremely rare. Therefore, we focused our literature review on cases of phlebosclerosis, phlebitis, and/or thrombophlebitis that resulted in restriction of elbow extension. In this study, we excluded cases originating from lymphatic vessels and considered only those caused by venous pathology. To the best of our knowledge, including the present case, only 5 cases have been reported to date (**Table 1**).^2,8–10)^

**Table:** Table 1 Phlebosclerosis, phlebitis, and thrombophlebitis causing elbow extension limitation

Case no.	Year	Author	Age	Gender	Etiology	Anticancer drugs	Symptoms of antecubital fossa or elbow	Patency of cutaneous vein	Histopathological findings	Treatment	Outcome
1	1963	Ashken^8)^	21	M	Axillary abscess	–	Pain, strings, and extension limitation	N/A	A small vein surrounded by fibro-fatty connective tissue	N/A	The bands disappeared 4 weeks later
2	1998	Saab^9)^	33	M	Blood donation	–	Pain, a tight band, and extension limitation to 70°	N/A	N/A	Physiotherapy and NSAIDs	The tight band disappeared while playing cricket
3	2008	Fujimaki^2)^	66	F	Chemotherapy	DOX CPA	Pain, subcutaneous cord and extension limitation	MR-venography; blood flow seemed barely maintained	The vessel cavity was almost totally occluded with granulation tissue	ROM exercise for 3 months failed → Surgical resection	The elbow was extended −10° at 9 months after surgery
4	2010	Hasegawa^10)^	71	F	Chemotherapy	EPI CPA 5-FU	A cord-like vein structure and extension limitation	US; a hypoechoic tubular structure without any Doppler signal	The vein was almost obliterated by a concentric fibrous thickening	Oral antibiotics and anti-inflammatory agents for more than 6 months failed→ Surgical resection	The elbow was fully extended after 1 week of postoperative rehabilitation
5	2025	Ishikawa	54	F	Chemotherapy	EPI CPA Tmab Pmab DTX	Two cord-like structures and extension limitation	US; blood flow within the lumen with Doppler signal	Partial thickening of the intima, loss of the smooth muscle layer, and proliferation of elastic and collagen fibers	Physiotherapy for 6 months failed→ Surgical resection	Complete extension at 5 months after venous resection

M: male; F: female; DOX: doxorubicin; CPA: Cyclophosphamide; EPI: Epirubicin; 5-FU: Fluorouracil; Tmab: Trastuzumab; Pmab: Pertuzumab; DTX: Docetaxel; N/A: not applicable; MR: magnetic resonance; US: ultrasonography; NSAIDs: non-steroidal anti-inflammatory drugs; ROM: range of motion

These cases involved 3 women and 2 men, with an age range of 21–71 years. In all cases, cord-like structures in the elbow veins caused elbow extension limitation. In the literature cited in **Table 1**,^2,8–10)^ the duration until contracture became apparent was approximately 2–3 weeks. The reported causes were venipuncture in 4 cases (3 for chemotherapy and 1 for blood donation) and infection in 1 case. The chemotherapeutic agents involved included cyclophosphamide in 3 cases and epirubicin in 2 cases.

Among the 4 cases where histopathological examination was performed—1 case with partial tissue resection (Case 1^8)^) and 3 cases with cord resection (Cases 3,^2)^ 4,^10)^ and 5)—3 were found to have cord-like structures caused by post-phlebitic scarring of the veins. Venous blood flow was preserved in only 2 cases, including Case 3^2)^ and our case (Case 5). In Case 1, the cord-like structure disappeared spontaneously, leading to symptom resolution.^8)^

A case has been reported in which thrombotic phlebitis developed in the trunk and axilla, leading to the formation of cord-like structures at the elbow associated with Mondor’s disease, resulting in extension dysfunction of the elbow joint (Case 4^10)^). The fibrous changes, hyalinization, and calcification of the vein due to phlebosclerosis are irreversible conditions. Therefore, in this case, conservative treatment did not lead to improvement, and the restriction of joint range of motion was progressive, necessitating surgical excision.

Regarding the chemotherapeutic agents implicated, cyclophosphamide and epirubicin were the most frequently reported,^2,10)^ both of which are known to have a high risk of inducing phlebitis.^1)^ Before administering such agents, patients should be informed about the risk of thrombophlebitis. Additionally, if vascular pain or other symptoms suggestive of vasculitis are observed, close monitoring and careful follow-up are essential. To minimize these risks, the administration of such chemotherapeutic agents should be performed through a central venous route, for example via an implanted subcutaneous port catheter, and the use of peripheral veins should be avoided.

## Conclusion

We presented a rare case of phlebosclerosis causing joint range of motion limitation in the elbow and reviewed previously reported cases. The appearance of cord-like structures following chemotherapy should prompt consideration of phlebosclerosis, and surgical resection of the venous cords should be considered when joint mobility impairment occurs.

## Declarations

### Consent for publication

Consent for publication was obtained from the patient.

### Ethics approval

This study was approved by the Ethics Review Committee of Hamamatsu University School of Medicine (Approval No. 17-129).

### Disclosure statement

The authors declare that there is no conflict of interest.

### Author contributions

Study conception: NI and NU

Patient management: NI, NU, KI, MS, KK, HTs, and YE

Data collection: NI

Manuscript preparation: NI and NU

Critical review and revision: all authors

Final approval of the article: all authors

Accountability for all aspects of the work: all authors.
